# The G127V variant of the prion protein interferes with dimer formation in vitro but not in cellulo

**DOI:** 10.1038/s41598-021-82647-w

**Published:** 2021-02-04

**Authors:** Sudheer Babu Sangeetham, Anna Dorothee Engelke, Elfrieda Fodor, Sarah Laura Krausz, Jörg Tatzelt, Ervin Welker

**Affiliations:** 1 Institute of Biochemistry, Biological Research Centre, Szeged, 6726 Hungary; 2 Doctoral School of Multidisciplinary Medical Sciences, University of Szeged, Dugonics square 13, Szeged, 6720 Hungary; 3 Department Biochemistry of Neurodegenerative Diseases, Institute of Biochemistry and Pathobiochemistry, Ruhr University Bochum, 44801 Bochum, Germany; 4 Institute of Enzymology, Research Centre for Natural Sciences, Budapest, 1117 Hungary; 5 School of Ph.D. Studies, Semmelweis University, Budapest, 1085 Hungary; 6 Aktogen Hungary Ltd., Kecskemét, 6000 Hungary; 7 Cluster of Excellence RESOLV, Bochum, Germany; 8 Present Address: Department of Neurology, Medical Faculty, Heinrich-Heine-University Düsseldorf, 40225 Düsseldorf, Germany

**Keywords:** Prion diseases, Prions

## Abstract

Scrapie prion, PrP^Sc^, formation is the central event of all types of transmissible spongiform encephalopathies (TSEs), while the pathway with possible intermediates and their mechanism of formation from the normal isoform of prion (PrP), remains not fully understood. Recently, the G127V variant of the human PrP is reported to render the protein refractory to transmission of TSEs, via a yet unknown mechanism. Molecular dynamics studies suggested that this mutation interferes with the formation of PrP dimers. Here we analyze the dimerization of 127G and 127VPrP, in both in vitro and a mammalian cell culture system. Our results show that while molecular dynamics may capture the features affecting dimerization in vitro, G127V inhibiting dimer formation of PrP, these are not evidenced in a more complex cellular system.

## Introduction

Transmissible spongiform encephalopathies (TSE) are rare neurodegenerative fatal diseases, occurring as Creutzfeldt-Jakob diseases (CJD, including sporadic, iatrogenic, variant, and familial/genetic CJD)^1–4^, Gerstmann-Sträussler-Scheinker syndrome (GSS)^1,5^, fatal familial insomnia (FFI)^1,3,6^ and kuru^1,7,8^ in human, scrapie in sheep^9^ and bovine spongiform encephalopathies (mad cow disease) in cattle^10^. TSEs typically occur spontaneously or due to transmission by diseased individuals and less often due to inherited mutation in the PRNP gene that encodes for the prion protein. All these conditions ultimately are caused by the conformational conversion of the normal cellular prion protein (PrP^C^), a cell surface glycosylphosphatidylinositol (GPI) anchored membrane protein, to a protease-resistant, insoluble scrapie conformation (PrP^Sc^) with increased β-sheet content. PrP^Sc^ is thought to be both an essential part of the transmissible agent of the disease and the cause of the pathological changes, such as rapid neurodegeneration^1,11,12^.

PrP has been associated with several physiological processes since its discovery, but its most concrete function by now discerned at molecular level, has only recently been revealed^13^.

It is well established the impact that prion diseases had in the past and present on humans^14–16^. The widespread incidence in the past of the prion disease kuru in Papua New Guinea, originated as a result of the ritualistic anthropophagy of human CNS-derived tissue among the people of the Fore tribes, evolving to become the prime cause of death in this region up until the middle of the twentieth century^17,18^. It has been found that this incident of spread of kuru at the same time also resulted a parallel spread of a resistant genotype among the people of the Fore tribes: the G127V variant in association with M129V, playing protective role against kuru^19^. Interestingly, transgenic mice expressing only human V127PrP were completely resistant to all prion strains, demonstrating a different molecular mechanism to that exerted by M129V, which provides a protection against classical CJD and kuru in the heterozygous state, but not against variant CJD^20^.

The mechanism of PrP^Sc^ formation that is critical for disease development is not entirely clear. In order to understand this process, a number of efforts have been made to develop conversion reactions, employing either brain-derived mammalian PrP^C^ or bacterially expressed recombinant PrP. These recapitulated several features of the conversion, such as the proteinase K (PK)-resistance of the infectious material or the necessary co-factors, providing some insights to the process^21–35^. In vitro spontaneous conversion of PrP is generally attempted by applying chemicals and/or varying the pH or temperature in order to enhance the process and to obtain forms of PrP that are at least in some aspects reminiscent to PrP^Sc^^24,36–42^. In this line, the reduction of the single disulfide bond present in the PrP’s structure, destabilizes the protein^43,44^, while also promotes its conversion to oligomeric forms^39,44,45^. These misfolded oligomers are β-sheet rich and exhibit low-level PK-resistance, however, they are likely of different structures than those of PrP^Sc^ derived from brain, which possess an intact intramolecular disulfide bond^46,47^. However, templated in vitro conversion is much more successful in generating authentic PrP^Sc^ material^23,28^ although infectious PrP^Sc^ formation is reported in non-templated conversion as well^22,23,29,48^.

The effect of the G127V mutation has been studied on the fibril formation kinetics of the mouse prion protein (mPrP), using an in vitro conversion assay, and important differences had been revealed between the wild type and the G126V mutant mPrP (correspondent of the G127V HuPrP), such as the critical concentration being higher, the lag phase being longer and the initial effective rate constant of fibril growth being slower in the case of the mutant^49^.

While these results provide a potential biophysical explanation for the observed protecting effect of G127V against prion diseases, a structural explanation is still missing. Recently, the structural and dynamic features of G127V variant were determined by NMR^50^, which together with molecular dynamics simulations suggested that HuPrP(G127V) prevents the formation of PrP dimers, offering a potential explanation for the inhibition of PrP^Sc^ formation by the G127V mutation^50,51^.

The role of dimer formation in PrP^Sc^ is not fully elucidated. A number of studies employing various approaches suggested that in in vivo conditions, at least some fraction of PrP^C^ is existent as alpha helical dimers^52–55^ evoking an importance of dimerization in PrP^C^’s physiological functions, importantly also in the cytoprotectivity played by PrP^C^^56^. It has also been demonstrated by using N2a cells that the hydrophobic domain (aa 112 through 133) plays an essential role in the ability of the mouse PrP to dimerize^54^. The fragment aa 90 through 231 of the recombinant Syrian hamster PrP [PrP(90–231)] was also found to form alpha helical dimers in the presence of low SDS concentrations^57^, although, it was not confirmed whether this would represent the native form or if it would already be an intermediate or a species in the conversion pathway of PrP^58,59^. It can be envisaged that dimer formation is critical at the beginning of PrP’s misfolding and this may or may not involve at first the partial unfolding of PrP.

To test the effect of G127V on the dimerization of the prion protein we employed two approaches: a mammalian cell culture based approach that follows the formation of mPrP dimer through co-immunoprecipitation of the dimers in presence and absence of an intermolecular disulfide bond^60^ and an in vitro system that employs recombinant mouse prion protein containing a genetically encoded crosslinkable *p*Bpa residue^61^. Our in vitro results are in line with the contention that was put forward in molecular dynamics studies^50,51^ that the G127V mutation inhibits the formation of PrP dimers, however the cell culture experiments do not seem to display similar effects/observations.

## Results

### In vitro crosslinking

#### mPrP variants with site specifically inserted pBpa can be used to study heterodimerization

**Figure 1:**
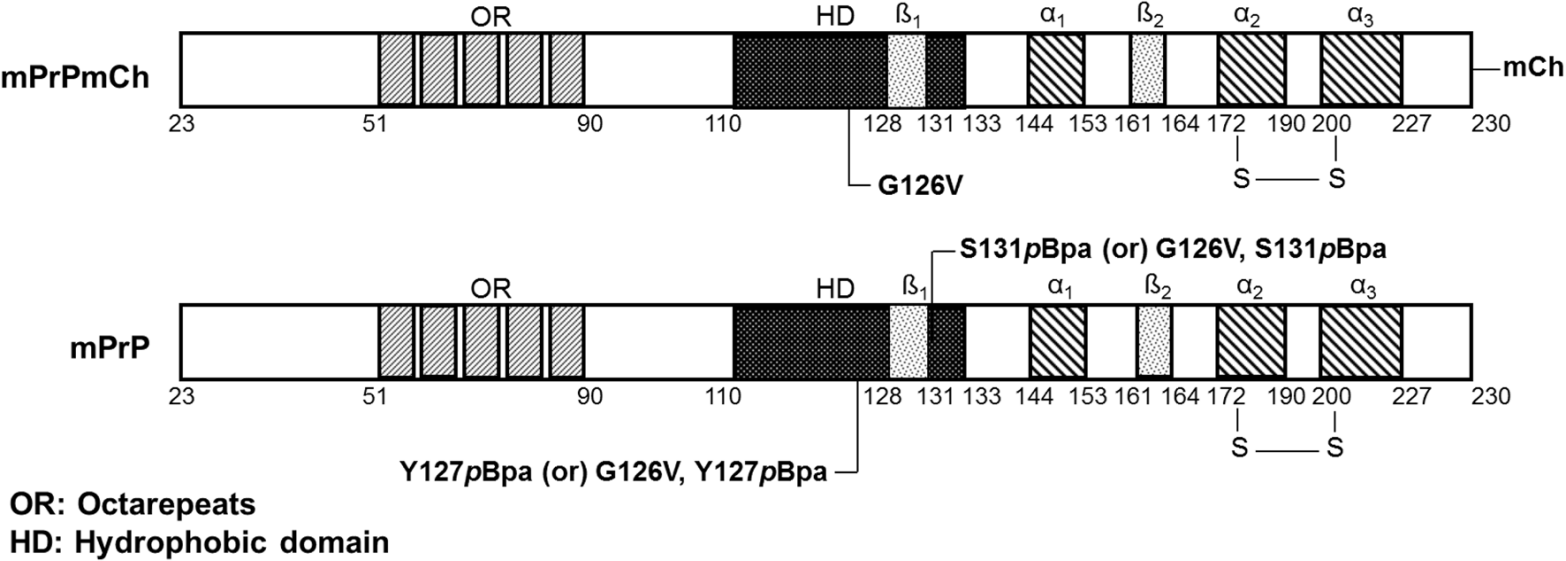
Schematic illustration of the recombinant prion proteins used for testing the hetero- and homodimerization of mPrP. Top scheme represents the structure of the mouse prion protein tagged with an mCherry (mCh), mPrP-mCh, with indication of the location for the G126V mutation. Lower scheme depicts the untagged mPrP, indicating the mutations used, *p*Bpa incorporation (at either position 127 or 131) and/or G126V.

**Figure 2:**
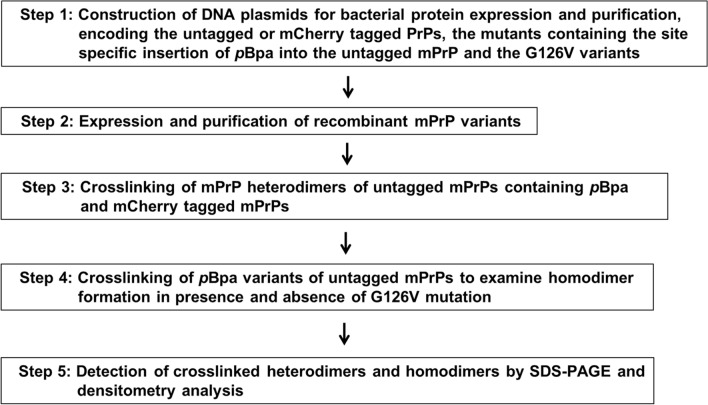
The major steps of the procedure employed to investigate the effect of G126V mutation on the heterodimer and homodimer formation of mPrP in in vitro conditions. DNA plasmids were constructed for *E. coli* expression of wild type, G126V and *p*Bpa mutants mPrPs with or without fusion with mCherry (Step 1). The purified proteins (Step 2) were selected in pairs with one possessing an mCherry tag and another an untagged mPrP that usually contained the *p*Bpa insertion to study heterodimer formation by crosslinking (Step 3). Single protein variants with *p*Bpa were also selected to study homodimer formation (Step 4). Crosslinked hetero- and homodimers were assessed by SDS-PAGE and densitometry analysis (Step 5).

The formation of a dimer by the bacterially expressed recombinant mouse prion protein has been demonstrated and the dimer interface has been mapped in our earlier work^61^ by using a genetically incorporated unnatural amino acid, *p*Bpa, that crosslinks to C-H groups of the protein backbone and side chains of nearby proteins situated within a distance of less than 3.1 Å, when irradiated by UV light. Here we used this system to investigate the effect of the G126V mutation (the mouse equivalent of the human G127V mutation) on the formation of a prion dimer. The *p*Bpa is incorporated either into the wild type (WT) or into the G126V mutant prion protein’s sequence and to specifically monitor heterodimerization, an mCherry-tag is fused to one of the mPrPs in order to be able to distinguish the interacting proteins (Fig. 1), i.e. WT and the mutant form, by SDS-PAGE. Effective crosslinking of mPrP dimers was obtained earlier with 127*p*Bpa positional mutation^61^, accordingly, to monitor the formation of the heterodimers we placed a 127*p*Bpa mutation into the sequence of the untagged mPrP in order to mediate covalent crosslinking to mPrP-mCherry fusion proteins (Fig. 2).

The configuration where the *p*Bpa is incorporated into the mPrP-mCherry fusion protein was not used for heterodimerization studies because upon UV irradiation a band appeared at the expected position of a heterodimer on the SDS-gel of the protein alone, mPrP(Y127*p*Bpa)-mCherry, without the presence of untagged mPrP as its binding partner.

#### Gly126Val mutation diminishes PrP dimerization

**Figure 3:**
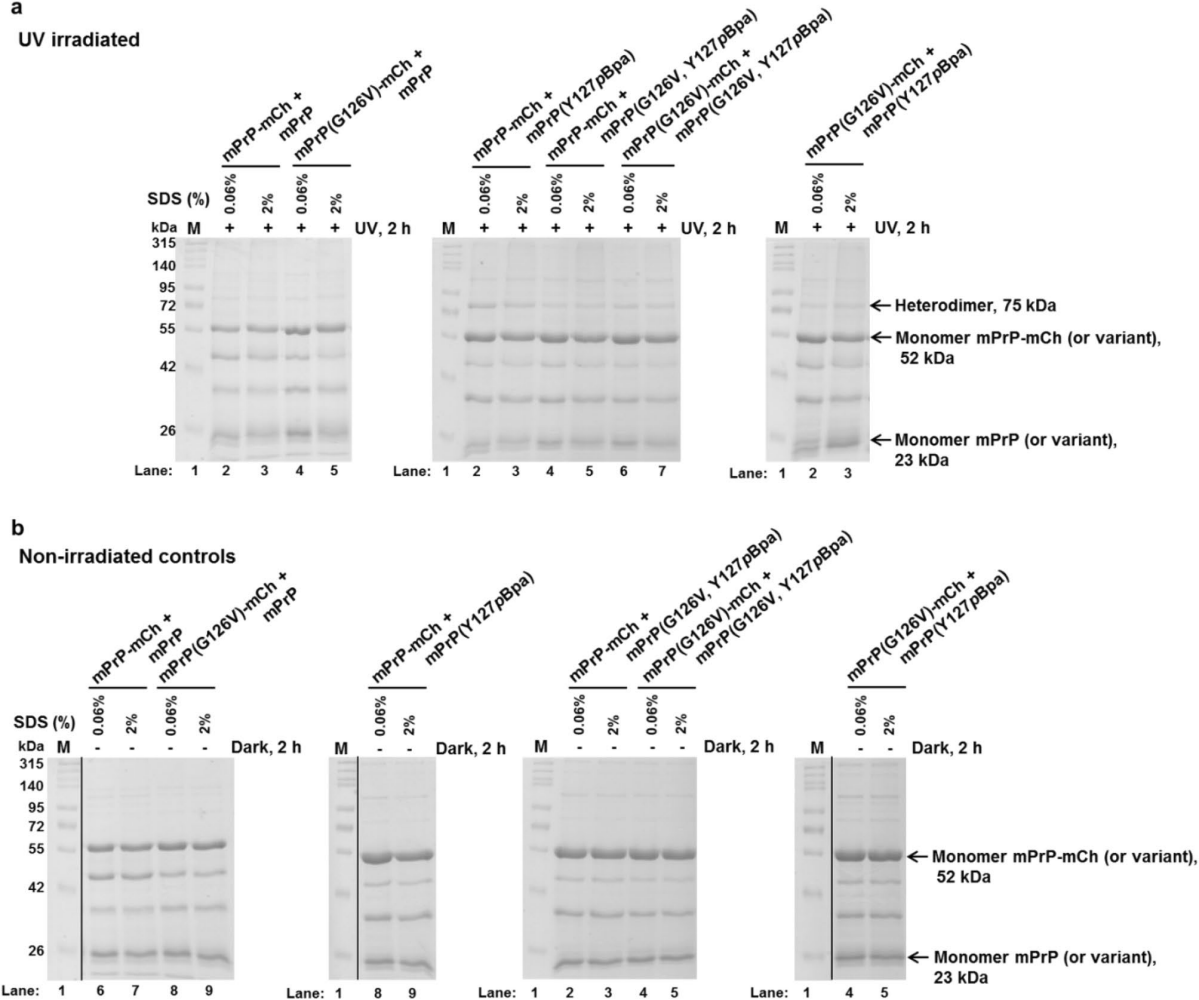
Crosslinked heterodimers of prion protein. Representative SDS-PAGE gel pictures of the photo-crosslinked reaction mixtures of various mPrP (“UV irradiated”, + UV) (**a**) and their control, non-irradiated (“Non-irradiated controls”, Dark) (**b**) counterparts. In case of all reaction-mixtures presented, *p*Bpa is present in the untagged mPrP at the position 127 (Y127*p*Bpa). The partner protein in each case is a prion-mCherry fusion protein with or without possessing a G126V mutation in the sequence of the prion. The reaction mixtures irradiated at 365 nm (+) and non-irradiated (−) for 2 h, are in presence of either 0.06% or 2% SDS in order to promote dimerization or to assess the background non-specific association of the proteins, respectively. The expected positions of the two kinds of monomers (the untagged mPrP and/or its mutant variants at 23 kDa, and of the mPrP-mCherry fusion protein and/or its valine mutant variant at 52 kDa) and of their heterodimer (75 kDa) is indicated on the figure. Lanes 1 (“M”) contain the same molecular weight marker loaded. The two bands appearing at 28 kDa and 46 kDa on the gels are cleavage products, originating from the partial hydrolysis of the main-chain acylimine linkage of the chromophore of mCherry, a phenomenon known to occur for DsRed-like chromophores upon sample-treatment, such as boiling in presence of SDS and dithiothreitol for SDS-PAGE analysis^72^. The vertical black lines delineate the position of cropping, where lanes unrelated to the figure were removed from the gel pictures (panel **b**). The images of the full-length gels are provided in Supplementary Fig. S2).

**Figure 4:**
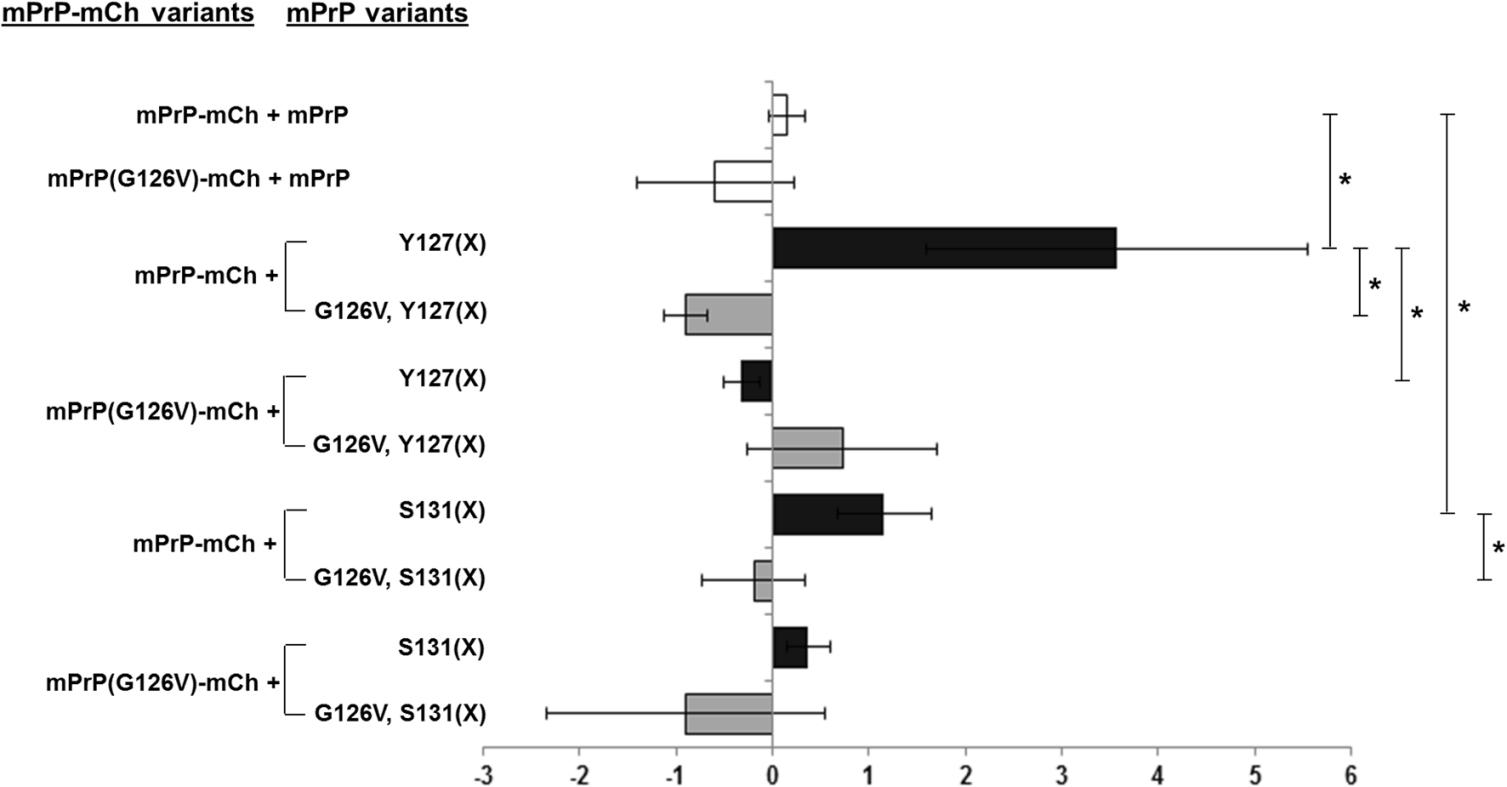
Diminished heterodimer formation in case of a G126V mutation in mPrP. The amount of crosslinked heterodimers are assessed for the indicated mCherry-tagged and untagged prion protein mixtures, using two different positions for the *p*Bpa (denoted as “(X)”) substitution (Y127*p*Bpa and S131*p*Bpa) in untagged mPrP. The values represent percentages calculated based on gel densitometry analysis as described in Materials and Methods. White bars indicate values obtained in case of control samples containing no *p*Bpa. Black and grey color bars indicate the % of heterodimers without and with Val mutation at position 126, respectively, in either mPrP or mPrP-mCh proteins. Statistical significances of the differences were calculated by unpaired t-test. Significantly different pairs are marked on the graph, and significance levels are indicated by stars, *p ≤ 0.05. “(X)” stands for *p*Bpa in the notation of Y-axis category names referring to the *p*Bpa-mutation at the indicated positions.

The mPrP(Y127*p*Bpa) effectively crosslinks mPrP-mCherry when the position 126 contains the WT Gly residue (Figs. 3 and 4). However, the presence of a Val in position 126 in mPrP or in mPrP-mCherry or in both of them at the same time, diminishes the appearance of heterodimeric mPrP(Y127*p*Bpa)—mPrP-mCherry crosslinked product, suggesting an inhibition upon heterodimer formation (Figs. 3 and 4). Next, we placed the *p*Bpa residue to another position and performed crosslinking experiments with mPrP(S131*p*Bpa) using the same experimental setup (Supplementary Fig. S1). *p*Bpa in the 131st position mediated significant, but less efficient crosslinkage between mPrP and mPrP-mCherry with Gly126. Nevertheless, Val126 placed to either mPrP or to mPrP-mCherry diminished the formation of the heterodimers (Fig. 4 and Supplementary Fig. S1) crosslinked by this positional mutant.

**Figure 5:**
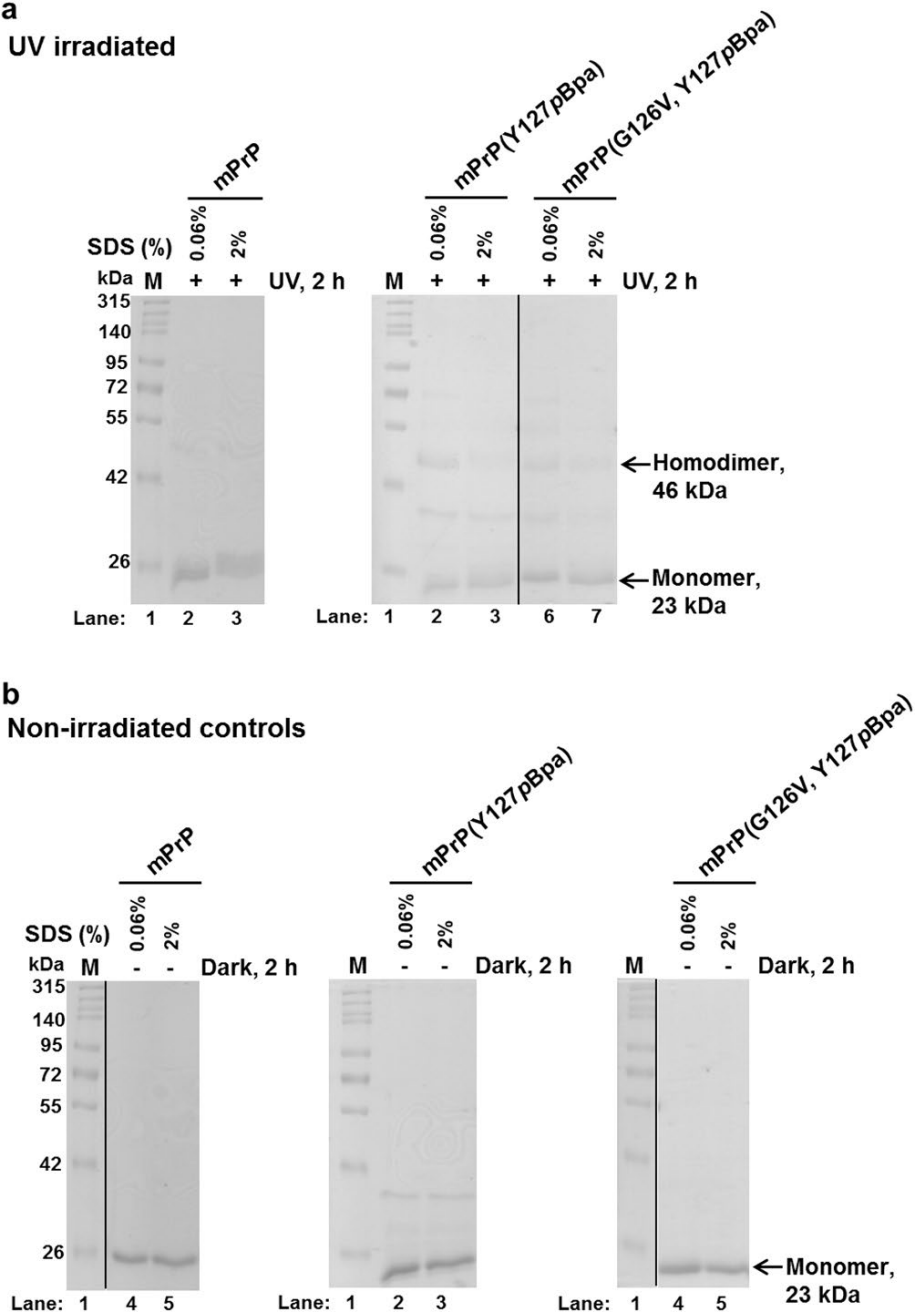
Effect of G126V mutation on mPrP homodimer formation. Representative SDS-PAGE gel pictures of crosslinked (“UV irradiated”, + UV) (**a**) and corresponding non-irradiated control (“non-irradiated controls”, Dark) (**b**) samples of single proteins. Untagged mPrPs with *p*Bpa mutation at position 127 were utilized to crosslink the homodimers formed in the absence or presence of a G126V mutation. The expected positions of monomers and homodimers are indicated. Lanes 1 (“M”) contain the same molecular weight marker. The vertical black lines on some of the gels delineate the position of cropping, where lanes unrelated to the figure were removed from the gel picture for an easier understanding. The images of the full-length gels are provided in Supplementary Fig. S2). Proteins (6 μM) are UV irradiated or incubated at dark, side-by-side in the presence of either 0.06% or 2% SDS (in PBS, pH 7.4), conditions that favor either dimerization or the monomeric form of the prion protein, respectively.

**Figure 6:**
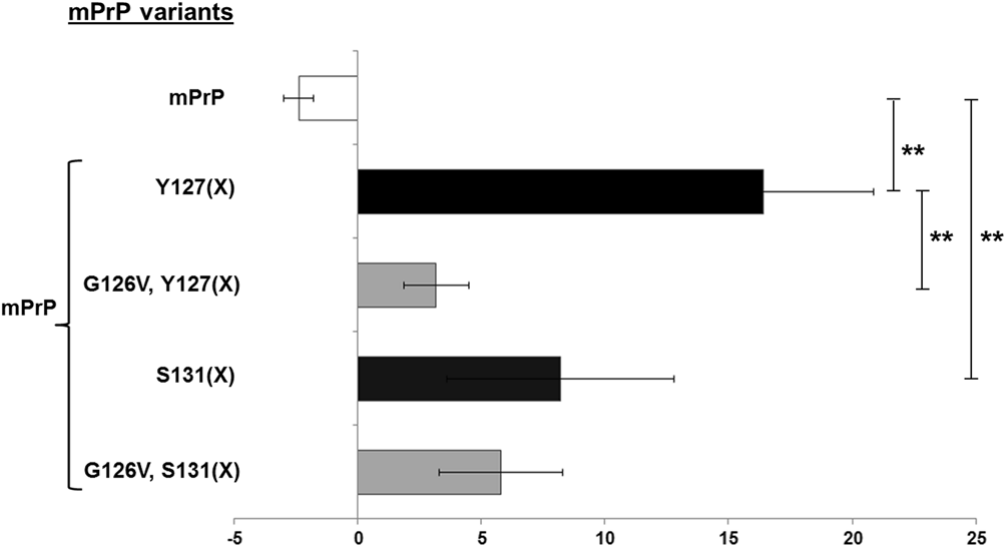
Homodimer formation is diminished for G126V mutants of mPrPs. The homodimers were detected by photo-crosslinking single-type *p*Bpa-mutant mPrPs: mPrP(Y127*p*Bpa) or mPrP(S131*p*Bpa) either comprising (grey color bars) or not (black bars) a 126Val mutation. mPrP containing no *p*Bpa was used as the negative control (white bar). Percentages were calculated based on gel densitometry analysis as described in the Materials and methods. Statistical significance of differences was calculated by unpaired t-test. Significantly different pairs and the level of significance are indicated on the graph, stars correspond to p levels **p ≤ 0.01. “(X)” stands for *p*Bpa in the notation of Y-axis category names referring to the *p*Bpa-mutation at the indicated positions.

We also examined the effect of the G126V mutation on the homodimerization of mPrP (Fig. 5, Supplementary Fig. S1). We employed three single *p*Bpa mutants, the Y127*p*Bpa, M128*p*Bpa and S131*p*Bpa prion variants, however, the diminishing effect of Val126 on homodimeric PrP formation reached a statistically significant level only in case of crosslinking via mPrP(Y127*p*Bpa), given the relative size of the scatter of the data of 3 parallel experiments with the other two mutants (Fig. 6).

### In cellulo crosslinking and native immunoprecipitation

#### V127 does not interfere with the formation of disulfide-bonded PrP dimers

**Figure 7:**
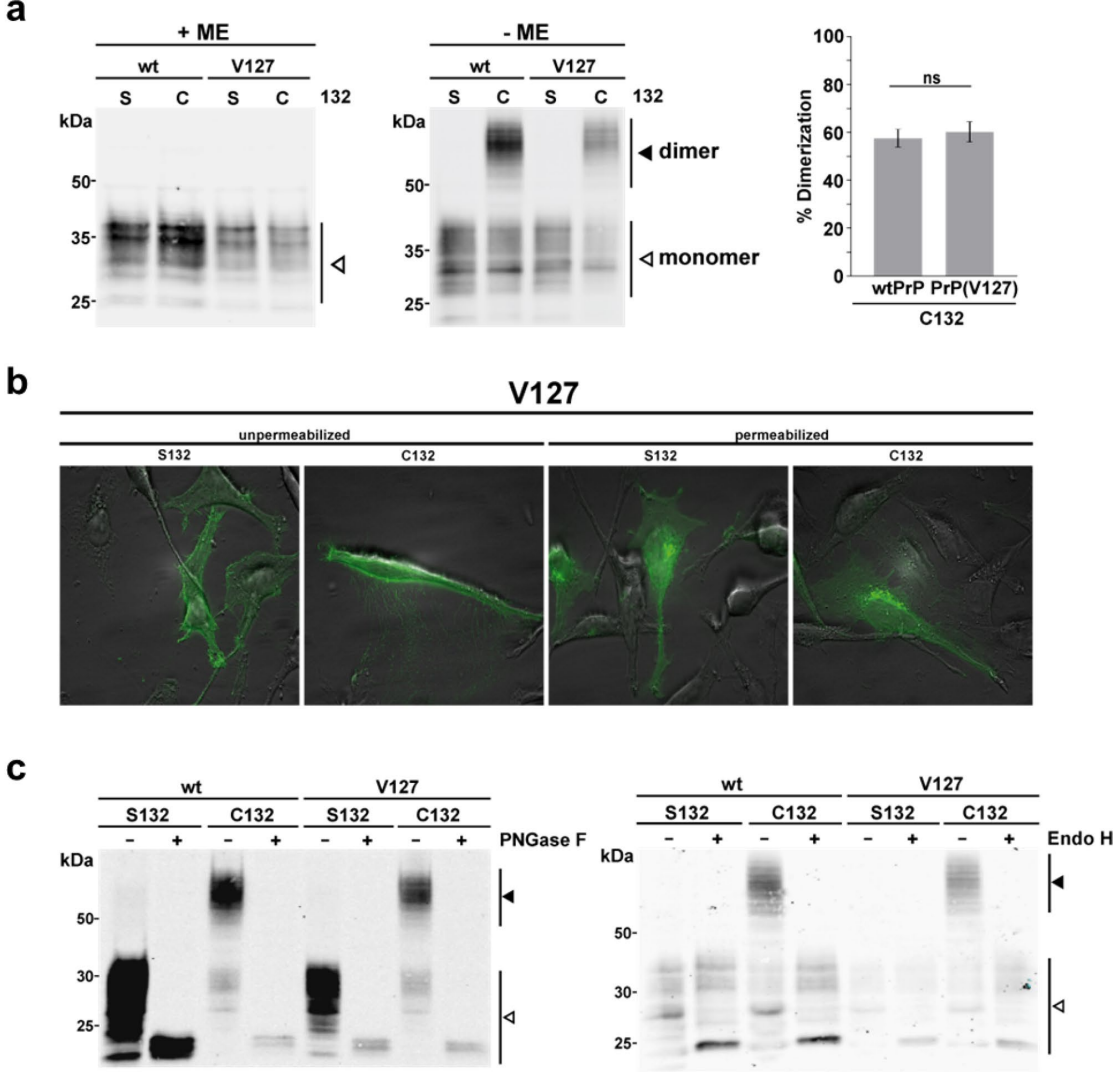
The V127PrP variant is complex glycosylated, located at the plasma membrane and forms homodimers similarly to G127PrP. (**a**) PrP forms disulfide bond-stabilized homodimers under non-reducing conditions in Western blot analysis. HeLa cells were transiently transfected with WTPrP (G127) or V127PrP containing a serine (S) or cysteine (C) at aa 132. The cysteine (C132) enables the formation of an intermolecular disulfide bond and allows separation of PrP dimers and PrP monomers on non-reducing SDS-PAGE. Cell lysates were prepared and denatured by boiling in Laemmli sample buffer with (+ ME) or without reducing agent (− ME). A white arrowhead represents monomeric PrP, the black arrowhead PrP homodimers. Right panel: quantifications of the dimerization efficiency measured densitometrically, *ns* not significant. Data represent mean ± SD of 4 independent experiments. (**b**) HeLa cells were transiently transfected with the V127PrP containing a serine (S132) or cysteine (C132) at aa 132 and were analyzed by indirect immunofluorescence. Fixed cells were either permeabilized or non-permeabilized and were detected with the 3F4 antibody. (**c**) Western blot analysis shows that dimerization of PrP does not interfere with complex glycosylation. HeLa cells were transiently transfected with the indicated constructs. To determine the glycosylation status lysates were treated either with peptide: N-Glycosidase F (PNGase F +, left panel) that cleaves high mannose, hybrid, and complex oligosaccharides from N-linked glycoproteins or endoglycosidase H, which cleaves only mannose rich oligosaccharides (Endo H +, right panel). Please note that the reaction buffer for PNGase F and Endo H contains a reducing agent, therefore only PrP monomers are seen in the PNGase F- and Endo H-treated samples. White arrowhead: monomer, black arrowhead: dimer.

**Figure 8:**
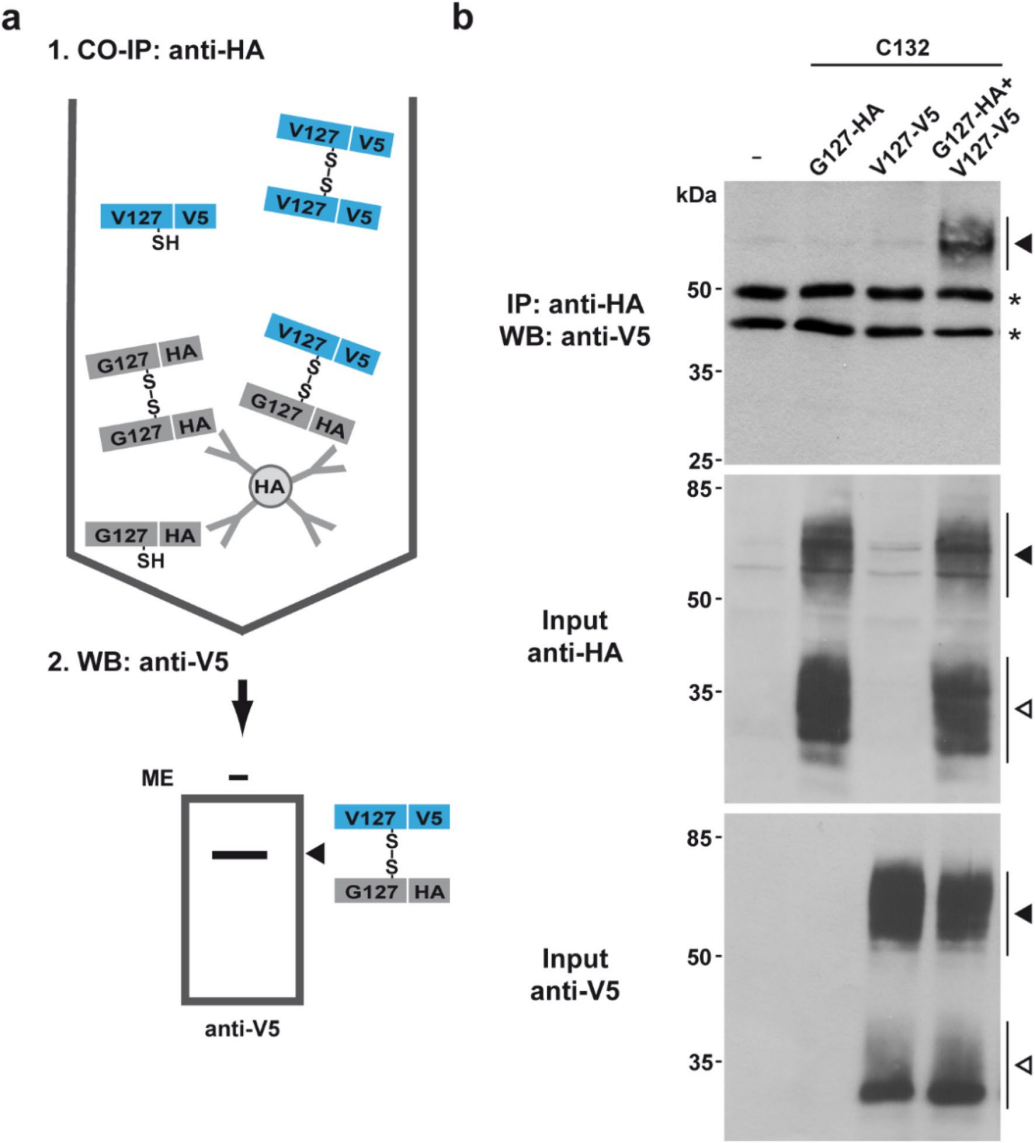
V127PrP forms disulfide bond-linked heterodimers with G127PrP. (**a**) Scheme of the experimental strategy to specifically detect G127/V127 heterodimers with disulfide bond stabilization. A cysteine was inserted as S132C mutation. HA-tagged G127PrP was first immunoprecipitated under non-reducing conditions using anti-HA agarose beads. In a second step Western blot analysis under non-reducing conditions with anti-V5 antibody revealed co-purified V5-tagged V127PrP in the immunopellet. The tags were inserted after aa 35 (**b**) HeLa cells were transiently transfected with either HA-tagged G127PrP, or V5-tagged V127PrP or both. Cells were lysed and HA-tagged WTPrP immunoprecipitated under non-reducing conditions with HA-agarose beads. The immunopellet was analyzed by Western blotting under non-reducing conditions using an anti-V5 antibody. Western blot analysis of the input controls is shown below. White arrowhead: monomer, black arrowhead: dimer. Asterisk: signal corresponds to primary antibody used in the IP.

Furthermore, we addressed homo- and heterodimerization of human V127PrP in a cellular context in (HeLa) cells. To relate easier, hereafter we use the numbering of aa positions corresponding to human prion sequence (note that aa 127 corresponds to aa 126 in the mouse prion sequence that we employed here). As described previously, PrP dimers can be stabilized by replacing serine 132 by cysteine. In case PrP dimerizes, an intermolecular disulfide bond can be formed that is stable in SDS buffer under non-reducing conditions^50,54^. After transient transfection of HeLa cells, the Western blot analysis reveals an additional signal at the size of PrP dimers in absence of the reducing agent β-mercaptoethanol (ME) (Fig. 7a, middle panel). A quantification indicated that V127 does not interfere with the formation of PrP homodimers (Fig. 7a, right panel). Under the conditions tested, about 60% of both G127- and V127PrP formed homodimers (Fig. 7a, right panel). Our previous studies revealed that forced dimerization does not interfere with maturation and cellular trafficking of PrP^60^. Similarly, V127PrP is located at the plasma membrane and is complex glycosylated (Fig. 7b,c). Next, we wanted to address the possibility that the G to V substitution interferes with the formation of G127PrP/V127PrP heterodimers. To this end we inserted an HA epitope tag into G127PrP and a V5 epitope tag into V127PrP. To allow intermolecular disulfide bond formation both proteins contain a cysteine at position 132 (Fig. 8a). After co-expressing G127PrP-HA and V127PrP-V5 in HeLa cells the lysates were subjected to an immunoprecipitation with anti-HA antibodies under non-reducing conditions. The immunopellet was then analyzed by Western blotting using anti-V5 antibodies. Indeed, V127PrP-V5 co-purified with G127PrP-HA, indicative of the formation of PrP heterodimers (Fig. 8b).

#### V127 does not interfere with the formation of PrP dimers in a native immunoprecipitation assay

**Figure 9:**
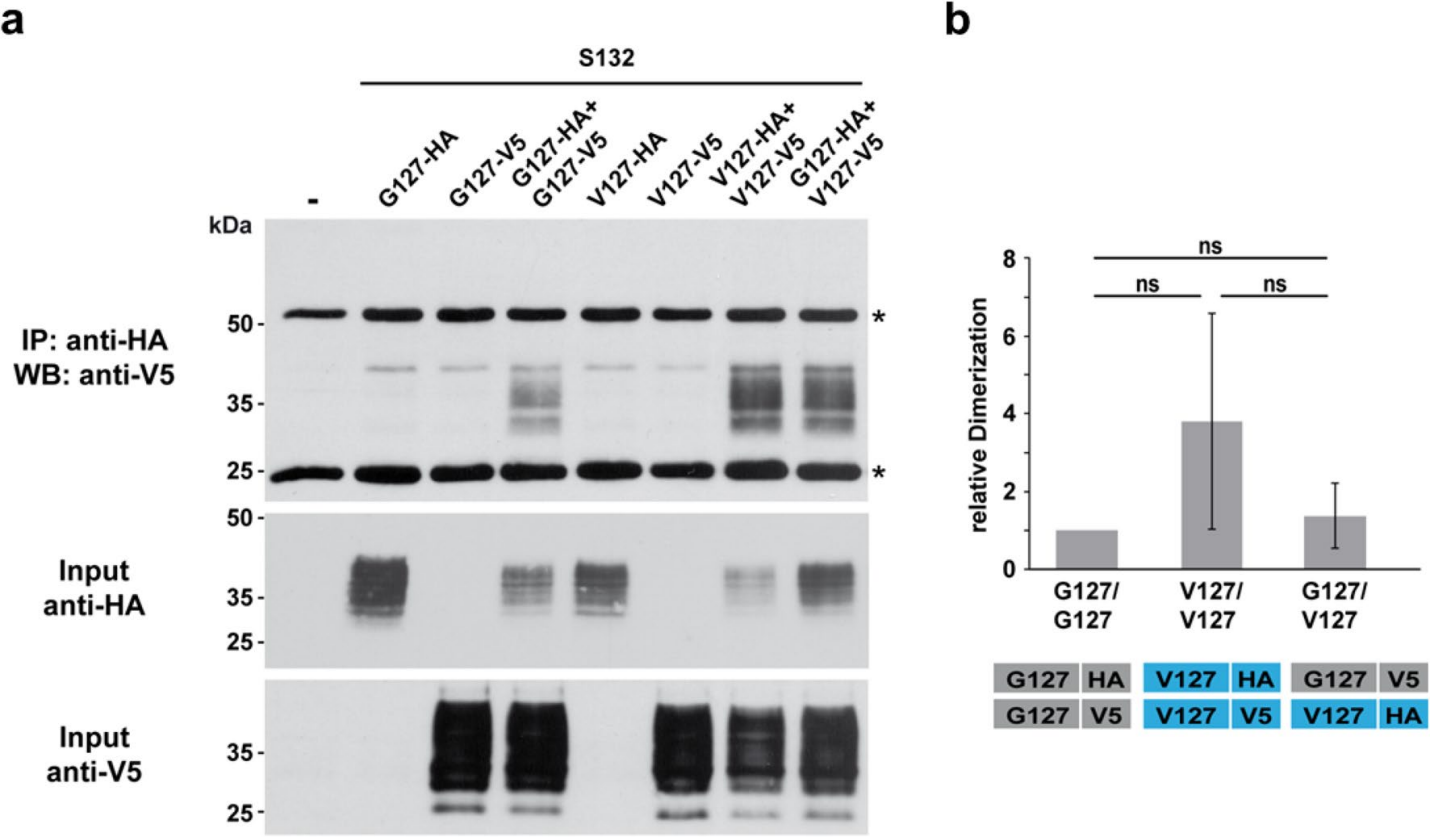
V127PrP forms homodimers and heterodimers with G127PrP under physiological conditions. (**a**) The serine variants of G127PrP and V127PrP were modified with an HA (G127PrP-HA; V127PrP-HA) or a V5 tag (G127PrP-V5; V127PrP-V5). The tags were inserted after aa 35. HeLa cells were transiently transfected with the indicated constructs. Transfected cells were lysed and HA-tagged PrP was immunoprecipitated in the absence of reducing agents with HA-agarose beads. The immunopellet was dissolved in Laemmli sample buffer containing β-mercaptoethanol and analyzed by Western blotting using an anti-V5 antibody. Western blot analysis of the input controls is shown below. Asterisk: signal corresponds to primary antibody used in the IP. (**b**) Quantification of the dimer formation measured densitometrically, as ratio of the dimer fraction to the HA-tagged PrP input control. WT/WT homodimers ratio was set as 1, *ns* not significant. Data represent mean ± SD of 3 independent experiments.

To assess the dimerization under physiological conditions, we performed native immunoprecipitation assays with HA- and V5-tagged PrP constructs, which lacks the Ser-to-Cys mutation (Fig. 9). Cells were co-transfected with an HA- together with a V5-tagged PrP construct and cell lysates were processed to an immunoprecipitation under native conditions with anti-HA antibody conjugated beads. A Western blot analysis of the immunopellet using anti-V5 antibodies verified the formation of homodimers of G127PrP and V127PrP as well of heterodimers under physiological conditions (Fig. 9a). Quantification of dimer formation as ratio to the input control showed no significant interference of the V127 mutation in the formation of PrP homo- or heterodimers (Fig. 9b).

In sum, the cell culture experiments indicate that the G to V substitution does not interfere with the dimerization of complex glycosylated and GPI-anchored PrP in cells.

## Discussion

Molecular dynamics studies suggest that Tyr127 (128 in the human prion protein) has a key role in dimer formation and altering the 126Gly to Val changes the orientation of the Tyr side chain, while diminishing PrP dimerization. We found earlier that position 127 of recombinant mPrP when harboring a photo-crosslinkable amino acid, *p*Bpa, and after UV irradiation results in the highest crosslinking for the Y127*p*Bpa variant among 24 single *p*Bpa-mutant variants tested^61^, confirming its key role in the formation of mPrP dimer interface and indicating at the same time that a Tyr to *p*Bpa mutation does not diminish dimer formation. Since the *p*Bpa substitution here is exactly in this aforementioned position, the G126V mutation in the mPrP(Tyr127*p*Bpa) protein may affect directly the orientation of the *p*Bpa residue, hence its crosslinking, without affecting the dimerization itself. However, the effect of Val126 is apparent in the crosslinking experiments of mPrP(Y127*p*Bpa) with mPrP(G126V)-mCherry where the Val126 mutation is in the partner protein and can not directly affect the orientation of the *p*Bpa side chain.

Former studies have pointed to the similarity between dimers of the full-length prion proteins formed in vitro in a test tube and those formed under more physiological conditions of mammalian cell culture experiments^52,54–58,61^. Here the two systems seem to provide opposing results. While the presence of G126V diminishes dimer formation under in vitro conditions, this propensity of the G126V mutation is not apparent in cell culture experiments. The discrepancy observed may be due to the confinement of the GPI-anchored prion protein to a two-dimensional membrane surface in the cell culture experiment, in contrast to the recombinant protein that is freely moving in a solution in the test tube. Indeed, transgenic mouse models indicated that the C-terminal GPI anchor has a major impact on folding of PrP^C^. Expression of a secreted full-length PrP mutant (S231X) induces neurodegeneration in transgenic mice^62^. Moreover, brain extracts of both human patients expressing Y226X and mice expressing S231X contain infectious prions and transmit disease^62,63^. These findings indicate that the loss of the GPI anchor/membrane attachment favors spontaneous conversion of PrP^C^ into infectious and neurotoxic conformers. Hosszu et al. has very recently reported on investigating the effect of V127 mutation on the structure and stability of recombinant human 119–231PrP fragment lacking the 23–118 region^64^. This flexible N-terminal part of PrP, although being unstructured, when examined in vitro, it has been reported to take part in the dimerization interface of PrP^58,61^. In the lack of these interactions mediated by the 23–118 N-terminal tail, the destabilizing effects of V127 are not evident in the in vitro experiments of Hosszu et al. By contrast, the N-terminal PrP fragment has been reported to be involved in interactions with cellular partners^65^, which may constrain its conformation and thus may be responsible also for the apparent absence of the destabilizing effect of the V127 mutation on PrP dimers in cellulo.

Whatever the correct explanation may be, the CO-IP data clearly show that the effect of the Val126 mutation on dimerization of the full length mPrP evidenced by in vitro and molecular dynamics studies, is not becoming apparent within the complexity of the cellular context.

In conclusion, our study emphasizes the important role of the flexible N-terminal and the GPI anchor in the conformational (mis)folding of PrP and the limitations of in vitro and in silico approaches to study misfolding of anchorless PrP as a model for the formation of infectious prions.

## Materials and methods

All chemicals were purchased from Merck/Sigma-Aldrich unless indicated otherwise.

### DNA plasmids used for bacterial protein expression and purification

To produce the untagged and mCherry tagged wild type mPrP and the *p*Bpa-mutant variants Y127*p*Bpa, M128*p*Bpa and S131*p*Bpa of the untagged mPrP, the same DNA-plasmids were used as in a previous work^61^, named as pRSETB-mPrP-Cys, pRSETB-mPrP-mCh-6xHis-Cys, pRSETB-mPrP(Y127x)-Cys, pRSETB-mPrP(M128x)-Cys and pRSETB-mPrP(S131x)-Cys, respectively. Each of the plasmid constructs encoding for a G126V mutant mPrP (that corresponds to the G127V mutation of the human PrP sequence) was generated from the corresponding pRSETB plasmids of either untagged mPrP or the mCherry tagged mPrP encoding plasmids by using standard molecular biology techniques. Briefly, the plasmids were digested with NgoMIV and AfeI enzymes (Thermo Fisher Scientific) and the following oligonucleotides were inserted as indicated in Table 1.

**Table 1:** Oligonucleotides used for the construction of the mPrP variants with Val126 mutations.

mPrP variant	Oligonucleotides used
G126V, 127X	1: CCGGTGCTGTAGTGGGGGGCCTTGGTGTGTAGATGCTGGGGAGT
	2: ACTCCCCAGCATCTACACACCAAGGCCCCCCACTACAGCA
G126V, 128X	1: CCGGTGCTGTAGTGGGGGGCCTTGGTGTGTACTAGCTGGGGAGT
	2: ACTCCCCAGCTAGTACACACCAAGGCCCCCCACTACAGCA
G126V, 131X	1: CCGGTGCTGTAGTGGGGGGCCTTGGTGTGTACATGCTGGGGTAG
	2: CTACCCCAGCATGTACACACCAAGGCCCCCCACTACAGCA
G126V	1: CCGGTGCTGTAGTGGGGGGCCTTGGTGTGTACATGCTGGGGAGT
	2: ACTCCCCAGCATGTACACACCAAGGCCCCCCACTACAGCA

### Expression and purification of recombinant mPrPs

Wild type, G126V- and *p*Bpa-mutant variants of mPrP with and without an mCherry fusion tag, were expressed and purified as described^61^, briefly as follows. The plasmids encoding proteins lacking *p*Bpa [pRSETB-mPrP-Cys, pRSETB-mPrP-mCh-6xHis-Cys and pRSETB-mPrP(G126V)-mCh-6xHis-Cys] were transfected into *E. coli* BL-21(DE3) competent cells, while those aiming for insertion of *p*Bpa [pRSETB-mPrP(Y127x)-Cys, pRSETB-mPrP(M128x)-Cys, pRSETB-mPrP(S131x)-Cys, pRSETB-mPrP(G126V, Y127x)-Cys, pRSETB-mPrP(G126V, M128x)-Cys and pRSETB-mPrP(G126V, S131x)-Cys] into pre-transformed competent *E. coli* BL-21(DE3) cells comprising already a pEVOL-pBpF plasmid^66^. Single colonies of transfected cells were used to inoculate starter cultures in Luria–Bertani (LB) media containing the corresponding antibiotics and were grown for 16 h at 37 °C. 1% inoculum was added to 800 ml LB media to yield a starting OD_600_ of ~ 0.05, and cultures were grown in presence of corresponding antibiotics. Protein expressions were induced at OD_600_ of 0.6–0.8 by adding 1 mM IPTG alone for mPrP, mPrP-mCh and mPrP(G126V)-mCh or together with 0.02% arabinose and addition of 1 mM *p*Bpa, for the *p*Bpa-mutant PrPs. After induction, the cells were cultured for an additional 16 h, at 37 °C in case of the untagged mPrPs (WT and mutant proteins), while in case of mPrP-mCherry fusion proteins, for 4 h, at 37 °C. The bacterial cells were harvested and proteins were purified as described in^61^. The purified proteins eluted from the Ni–NTA columns were dialysed in three steps: first in 20 mM sodium acetate, pH 5.5, 1 mM EDTA and 1 mM EGTA, at a protein to buffer ratio of 1:1000 vol:vol, at 4 °C for 6 h, and at the second and third steps in 20 mM sodium acetate, pH 5.5, at 1:1000 vol:vol sample to dialysate ratio at 4 °C for 6 and 12 h, respectively. This buffer was used to store the proteins, either on ice or frozen at − 20 °C, until further use. The purity of the dialyzed proteins was tested on 10% SDS-PA gels with RAMA staining^67^ and protein concentrations were measured by Bradford method^68^.

### Photo-crosslinking of heterodimers and homodimers of mPrP

To study the hetero- or homodimerization of mPrP, photo-crosslinking was performed using protein variants containing *p*Bpa mutation, either alone or as being one of the interacting pairs, respectively. In order to examine the heterodimerization, mPrP-mCh or mPrP(G126V)-mCh was used and crosslinked with one of the following proteins: mPrP(Y127*p*Bpa), mPrP(G126V, Y127*p*Bpa), mPrP(S131*p*Bpa) or mPrP(G126V, S131*p*Bpa).

Homodimerization was examined by crosslinking of single variants: mPrP, mPrP(Y127*p*Bpa), mPrP(G126V, Y127*p*Bpa), mPrP(M128*p*Bpa), mPrP(G126V, M128*p*Bpa), mPrP(S131*p*Bpa) and mPrP(G126V, S131*p*Bpa). Photo-crosslinking and evaluation of the crosslinked products were done as described earlier in^61^. Briefly, the reaction mixtures of heterogeneous or single proteins were irradiated at 6 µM of total protein in presence of either 0.06% SDS or 2% SDS (to favor dimerization or to assess the nonspecific background crosslinking, respectively) in phosphate-buffered saline (PBS) (137 mM NaCl, 2.7 mM KCl, 6 mM Na_2_HPO_4_.2H_2_O, 1.4 mM KH_2_PO_4_, pH 7.4) for 2 h by 365 nm UV light. Reaction volumes of 100 µl in 1.5 ml microfuge tubes were placed on ice at a distance of 5 cm from the UV-tubes for crosslinking, while similar samples were kept at dark as controls. Samples were analyzed on 10% SDS-PA gels. The efficiency of heterodimer or homodimer formation of mPrP variants were measured densitometrically as described previously^61^, briefly as follows. The gels were scanned and the images were analyzed for band intensities by the ImageJ software version 1.52a^69^. To determine the percentages of the crosslinked heterodimer PrPs, the area corresponding to the heterodimer band on the intensity plot was divided by the total area corresponding to the sum of areas of all protein bands in the lane. The percentage of the homodimers formed was calculated as the area corresponding to the homodimer band divided by the sum of the monomer and the dimer area. All crosslinking experiments were performed three times, using independent protein samples (n = 3) obtained from different expressions. For data representations the mean ± SD of these values are used. The significance of the difference obtained for valine mutant and control was tested using unpaired Student's t-test. Asterisks represent as follows, *p ≤ 0.05 and **p ≤ 0.01.

### DNA plasmids used for mammalian cell transfection

Plasmids were amplified in *Escherichia coli* TOP10 (Thermo Fisher Scientific). The murine prion protein (GenBank accession number M18070) was modified by two amino acid exchanges (L108M/V111M)^70^ to enable detection by the monoclonal antibody 3F4^71^. The amino acid numbering that is indicated here refers to the human prion protein sequence. Mutations of the mouse prion protein were generated by standard PCR to insert an exchange from glycine at position 126, which corresponds to position 127 in the human prion sequence, to a valine (denoted hereafter G127 or V127PrP). For co-immunoprecipitation experiments the constructs were modified with a V5 tag (GGTAAACCGATACCGAACCCGCTCCTCGGTCTCGATTCGACG) or an HA tag (TACCCATACGATGTTCCAGATTACGCT) localized in the unstructured N-terminal region between amino acid 35 and 36. All mutated PrP constructs were inserted into pcDNA3.1/Neo (+) vector (Invitrogen).

### Antibodies and reagents

The following antibodies were used: anti-PrP monoclonal antibodies 3F4^71^, anti-HA (mAb, MMS-101R; Covance), mouse monoclonal anti-V5 antibody (mAb, R960CUS; Thermo Fisher Scientific), mouse monoclonal anti-GAPDH (mAb, AM4300; Thermo Fisher Scientific), horseradish peroxidase (HRP)-conjugated goat anti-mouse IgG (Thermo Fisher Scientific), IRDye conjugated secondary antibody (IRDye 800CW donkey anti-mouse; LI-COR Biosciences). All standard chemicals and reagents were ordered from Merck/Sigma-Aldrich if not otherwise indicated. The monoclonal anti-HA-agarose beads were purchased from Merck/Sigma-Aldrich and cOmplete Mini EDTA-free Protease Inhibitor Cocktail from Roche.

### Cell lines, transfection and lysis

Human HeLa cells were cultured in Dulbecco’s Modified Eagle´s Medium with GlutaMAX (DMEM medium, Thermo Fisher Scientific) with the addition of 10% fetal calf serum, 100 units/mL penicillin and 100 µg/mL streptomycin. They were cultivated in a humidified 5% CO_2_ atmosphere at 37 °C and authenticated by their phenotype. For transfection cells were grown on a 3.5 cm cell culture dish (Nunc, Roskilde, Denmark). Plasmid DNA was transferred into cells by lipofection with Lipofectamine LTX with Plus Reagent (Thermo Fisher Scientific/Life Technologies) according to the manufacturer`s instructions. After 24 h, cells were washed twice with cold PBS, harvested by scraping them off the plate and pelleted via centrifugation (5000*g*, 10 min). The cell pellet was resuspended in detergent buffer (0.5% Triton X-100, 0.5% sodium deoxycholate in PBS) for lysis. For deglycosylation cell lysates were treated with PNGase F or Endoglycosidase H (Endo H) for 1 h at 37 °C according to manufacturer’s instructions and afterwards analyzed by Western blotting. Immunofluorescence analysis was performed as described earlier^60^. HeLa cells were seeded on glass coverslips and transiently transfected with the indicated constructs. After 24 h cells were fixed with 4% paraformaldehyde for 10 min. One set was permeabilized with 0.2% Triton in PBS and the other set was left unpermeabilized. All cells were blocked with blocking solution (5% normal goat serum in PBS) for 1 h and incubated with the anti-3F4 antibody in blocking solution overnight at 4 °C. Cells were washed with PBS and incubated with the Cy3-conjugated anti-mouse secondary antibody (Alexa Fluor 488) for 1 h. After mounting with Fluoromount-G (Thermo Fisher Scientific), cells were visualized by fluorescence microscopy (Zeiss ELYRA PS.1 and LSM 880).

### Co-immunoprecipitation

To analyze formation of dimers, co-immunoprecipitation was performed as described earlier^60^, briefly as follows. HeLa cells were co-transfected with the indicated constructs (V5 or HA-tagged). Transiently transfected cells were lysed in a detergent containing buffer (0.5% Triton X-100, 0.5% sodium deoxycholate in PBS). Post nuclear supernatants were generated (14,000*g*, 10 min) and collected, and were incubated with anti-HA-agarose beads (overnight; 4 °C; rotating). The immunocomplex was washed twice with the lysis buffer and once with PBS before proceeding for analysis by Western blotting.

### Western blotting

Western blot analyses were performed as described earlier^60^. Briefly, cell lysates were boiled in Laemmli sample buffer with or without 4% β-mercaptoethanol. After protein separation using SDS-PAGE proteins were transferred to nitrocellulose by electroblotting. Membranes were incubated with blocking solution (TBS with 0.1% Tween 20 and 5% skimmed milk) for 1 h at RT and incubated with primary antibody in blocking solution for 18 h at 4 °C. The Western blots were washed with TBS-T (TBS with 0.1% Tween 20) and incubated with respective secondary antibody (IRDye-Infrared Technology, LI-COR Biosciences or horseradish peroxidase in TBS-T) for 1 h at room temperature. Protein signals were detected via the peroxidase activity and enhanced chemiluminescence using ECL Western Blotting Substrate (Promega) or by an ODYSSEY 9120 Scanner (LI-COR Biosciences).

### Quantification

Dimerization efficiency of the prion protein constructs was measured densitometrically (ImageJ software^69^ or Image Studio Lite from LI-COR Biosciences) and visualized as percentage of the dimer signal to the sum of monomer and dimer signal. In the case of immunoprecipitation the dimerization efficiency was calculated as ratio of the dimer fraction to the HA-tagged PrP amount in the input control normalized to the WT/WT homodimers ratio. Data represent mean ± SD (standard deviation). Statistical differences between two conditions were determined by using a Student´s t-test (*p < 0.05; ns: not significant).

## Supplementary Information

Supplementary Information
