# Supplementary material for: The G127V variant of the prion protein interferes with dimer formation in vitro but not in cellulo
Source: Sci Rep. 2021 Feb 4;11:3116. doi: 10.1038/s41598-021-82647-w (PMC7862613; doi:10.1038/s41598-021-82647-w)
Supplement: Supplementary file 1 — Supplementary Information [file 41598_2021_82647_MOESM1_ESM.pdf]

Supplementary Information for

The G127V variant of the prion protein interferes with dimer formation *in vitro* but not *in cellulo*

Sudheer Babu Sangeetham[1,2,+], Anna Dorothee Engelke[3,8,+], Elfrieda Fodor[1], Sarah Laura Krausz[4,5,6], Jörg Tatzelt[3,7,*] & Ervin Welker[1,4,*]

[1]Institute of Biochemistry, Biological Research Centre, H-6726 Szeged, Hungary.

[2]Doctoral School of Multidisciplinary Medical Sciences, University of Szeged, Dugonics square 13, H-6720 Szeged, Hungary.

[3]Department Biochemistry of Neurodegenerative Diseases, Institute of Biochemistry and Pathobiochemistry, Ruhr University Bochum, 44801 Bochum, Germany.

[4]Institute of Enzymology, Research Centre for Natural Sciences, H-1117 Budapest, Hungary.

[5]School of Ph.D. studies, Semmelweis University, H-1085 Budapest, Hungary.

[6]Aktogen Hungary Ltd, H-6000 Kecskemét, Hungary.

[7]Cluster of Excellence RESOLV, Bochum, Germany

[8]Present address: Department of Neurology, Medical Faculty, Heinrich-Heine-University Düsseldorf, 40225 Düsseldorf, Germany.

[+,*]These authors contributed equally to this work.

Correspondence to: Ervin Welker, Institute of Biochemistry, Biological Research Centre, Szeged, H-6726 Szeged, Hungary. Email: welker.ervin@brc.hu; Telephone: +36 30 4158500; Jörg Tatzelt, Department Biochemistry of Neurodegenerative Diseases, Institute of Biochemistry and Pathobiochemistry, Ruhr University Bochum, 44801 Bochum, Germany. Email: Joerg.Tatzelt@ruhr-uni-bochum.de; Telephone: +49 234 32 22429.

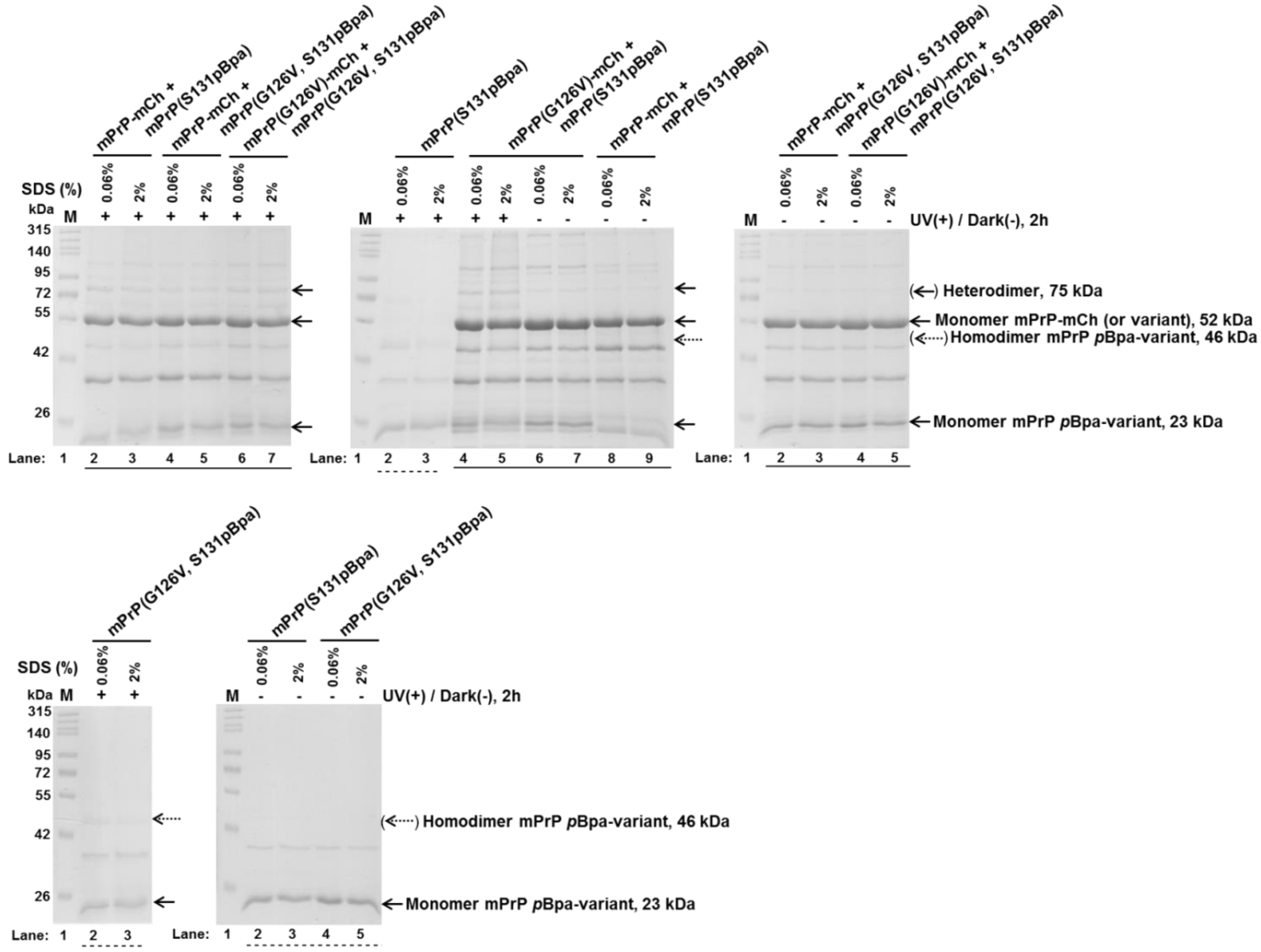

Supplementary Figure S1. mPrP heterodimers and homodimers, as detected by crosslinking the S131*p*Bpa mPrP variant. Representative SDS-PAGE gel pictures of the photo-crosslinked protein mixtures (lanes underlined by solid black lines) or single proteins (lanes underlined by dashed lines) of various mPrP ("UV irradiated", + UV) and their control, non-irradiated ("Non-irradiated controls", Dark) counterparts. *p*Bpa is present in the untagged mPrP (without mCherry) constructs with or without possessing a G126V mutation. The gels were used in calculations of the percent heterodimer presented on Fig. 4 and percent homodimer on Fig. 6, corresponding to the use of mPrP(S131*p*Bpa) variant. The protein mixtures or single variants are in presence of either 0.06% or 2% SDS in order to promote dimerization in the first case and to assess the background non-specific association of the proteins in the latter case, respectively. The expected positions of the monomers (untagged mPrP variants at ~23 kDa and mPrPmCh or its valine mutant variant at ~53 kDa), heterodimers and homodimers of untagged mPrPs are indicated on the figure by arrows. M: molecular weight marker.

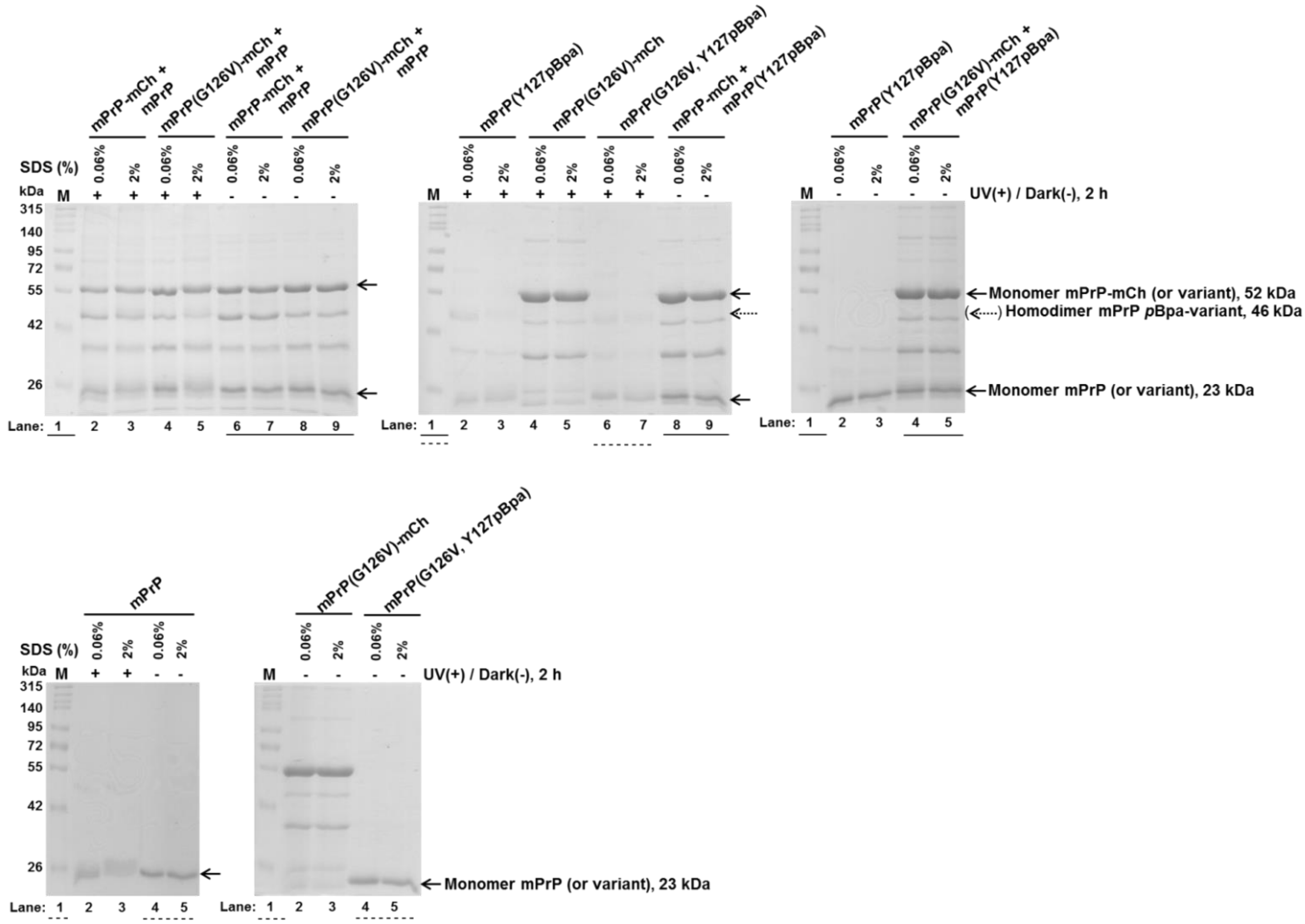

Supplementary Figure S2. Uncropped images of the representative gels of mPrP heterodimerization and homodimerization experiments. The lanes underlined by solid lines are presented as cropped images on Fig. 3b, showing the non-irradiated samples in the heterodimerization experiment. The lanes that are underlined by dashed lines were used as cropped images on Fig. 5a and b, to indicate homodimerization and their non-irradiated controls, respectively. The positions of the monomeric and homodimeric proteins are indicated by arrows.
